# Shifts in the eruptive styles at Stromboli in 2010–2014 revealed by ground-based InSAR data

**DOI:** 10.1038/srep13569

**Published:** 2015-09-01

**Authors:** Federico Di Traglia, Maurizio Battaglia, Teresa Nolesini, Daniela Lagomarsino, Nicola Casagli

**Affiliations:** 1 Dipartimento di Scienze della Terra, Università di Firenze Via La Pira 4, Firenze; 2 Dipartimento di Scienze della Terra, Università di Roma “La Sapienza”, Piazzale Aldo Moro 5, 00185, Roma, Italy; 3 Volcano Science Center, USGS, 345 Middlefield Rd, Menlo Park CA 94025, United States

## Abstract

Ground-Based Interferometric Synthetic Aperture Radar (GBInSAR) is an efficient technique for capturing short, subtle episodes of conduit pressurization in open vent volcanoes like Stromboli (Italy), because it can detect very shallow magma storage, which is difficult to identify using other methods. This technique allows the user to choose the optimal radar location for measuring the most significant deformation signal, provides an exceptional geometrical resolution, and allows for continuous monitoring of the deformation. Here, we present and model ground displacements collected at Stromboli by GBInSAR from January 2010 to August 2014. During this period, the volcano experienced several episodes of intense volcanic activity, culminated in the effusive flank eruption of August 2014. Modelling of the deformation allowed us to estimate a source depth of 482 ± 46 m a.s.l. The cumulative volume change was 4.7 ± 2.6 × 10^5^ m^3^. The strain energy of the source was evaluated 3–5 times higher than the surface energy needed to open the 6–7 August eruptive fissure. The analysis proposed here can help forecast shifts in the eruptive style and especially the onset of flank eruptions at Stromboli and at similar volcanic systems (e.g. Etna, Piton de La Fournaise, Kilauea).

Ground-Based Interferometric Synthetic Aperture Radar (GBInSAR) system offers an opportunity to image deformation due to shallow dike intrusions, shallow magma movement, opening/closing of eruptive fissures/ephemeral vents as well as landslides^1^,^2^,^3^,^4^,^5^,^6^ Since GBInSAR allows the user to choose the optimal radar location in terms of distance and incidence and azimuth angles, the deformation can be measured with exceptional geometrical resolution, while the very high sampling rate allows for continuous monitoring of the deformation^1^,^6^,^7^. Ground deformation at Stromboli, Italy, due to conduit processes may be difficult to detect and possibly related to strong explosions^8^,^9^,^10^ or syn-effusive deflations^11^. Both the 2002–2003 and 2007 flank eruptions completely drained the shallow storage system, as testified by the funnel-like vertical failure of the summit crater terrace associated with this eruption^11^,^12^,^13^,^14^,^15^,^16^,^17^,^18^ and to the lowering of the VLP source location^19^. The complete absence of strombolian activity is considered further evidence of the drainage of the shallower part of the Stromboli storage system^19^. According to ref. 11, the first two hours of the 2007 flank eruption drained 2.8**–**4.8 × 10^5^ m^3^ of magma. Evidence of new deep magmatic input, together with the rebuilding of the summit terrace, was observed beginning in late 2009^18^,^19^. During the 2010–2014 period, Stromboli experienced several episodes of intense volcanic activity^2^,^5^,^6^,^19^. Towards the end of May 2014, activity at Stromboli increased as the magma level inside the conduit rose. The volcano had several small effusions between June and August 2014 and peaked on August 7, 2014 with the opening of a fissure and an effusive eruption at ≈650 m a.s.l., ≈100 m below the NE the crater^20^.

## GBInSAR Data

**Figure 1:**
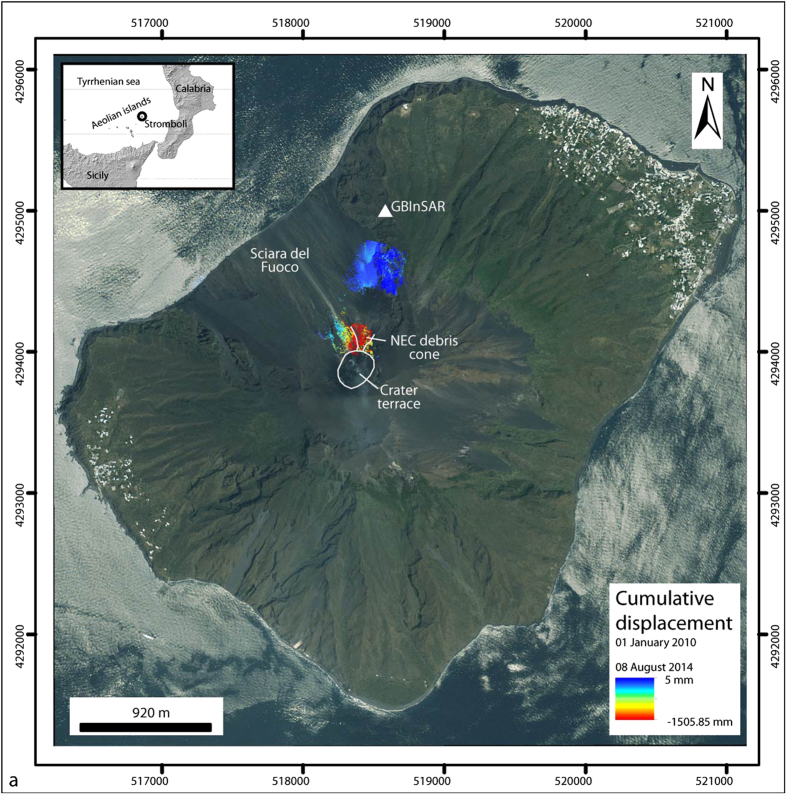
Displacement measured by GBInSAR at Stromboli. (**a**) Projection of GBInSAR cumulative displacement maps (1 January 2010—6 August 2014). The white triangle identifies the location of the instrument (in the insert, the location of Stromboli volcano is shown). The cumulated displacement maps is produced by the LiSA (Linear SAR) system produced by Ellegi LLC using proprietary GBInSAR technology by LiSALab LLC, a European Commission Joint Research Centre spin-off, and installed at Stromboli by the Dipartimento di Scienze della Terra—Università di Firenze (owner of the system), in the framework of the research agreements (SAR.net, SAR.net2, InGrID and InGrID2015 projects) with the “*Presidenza del Consiglio dei Ministri—Dipartimento della Protezione Civile*” (Presidency of the Council of Ministers—Department of Civil Protection). Map was generated using ESRI ArcGIS 8.2 platform.

The NE portion of the summit area of Stromboli has been continuously monitored since January 2003^6^ by a GBInSAR system located on a stable section of the flank, about 1.5 km away from the crater terrace (Fig. 1). GBInSAR Line Of Sight (LOS) is mostly sensitive to the N-S horizontal component of displacement (average azimuth angle = 15°). Negative and positive values of displacement indicate, respectively, a movement toward and away from the sensor. Given the location of the system (Fig. 1), flank instability could be a possible interpretation for movements towards the sensor but not for movements away from the sensor. Since we have observed both movements away and forward the sensor and the modelling indicates that the location and depth of the source are stable, we believe that the changes in the LOS correspond to either inflation (negative values – downslope movement toward the sensor) or deflation (positive values – upslope movement away from the sensor) of the summit area^1^,^2^,^3^,^4^,^5^,^6^.

**Figure 2:**
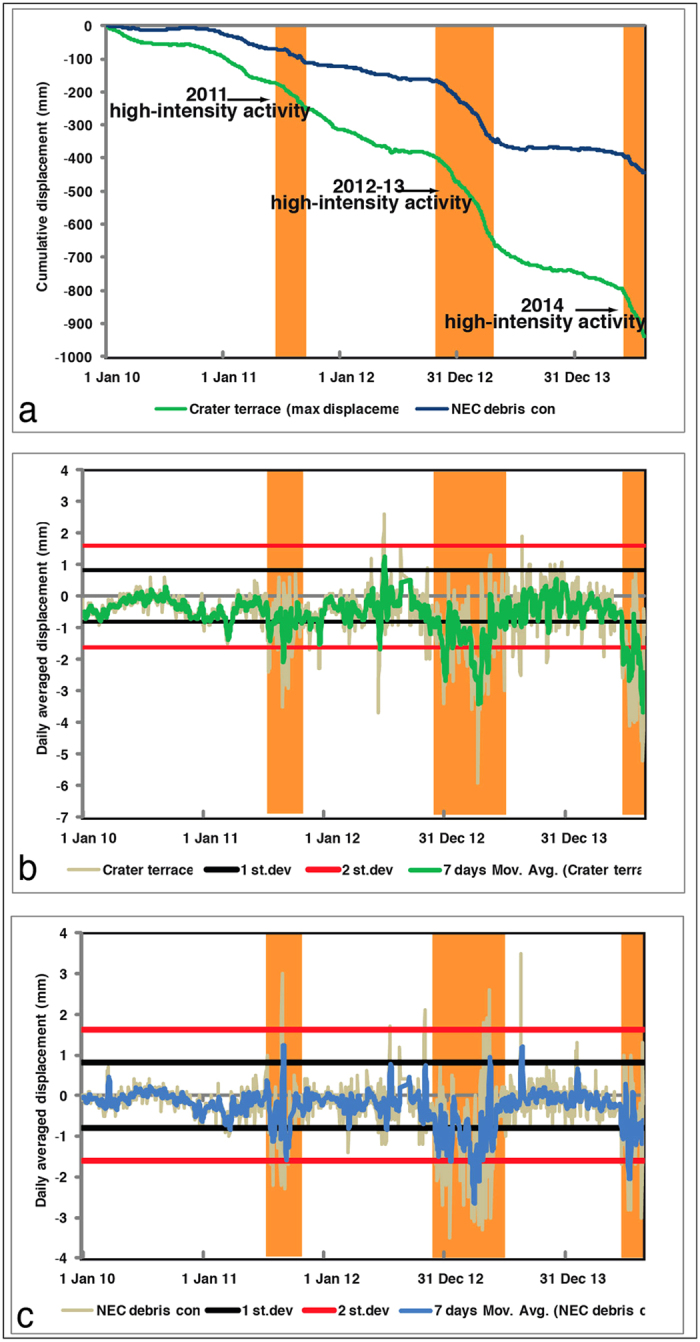
(**a**) GBInSAR cumulated time series of the largest displacements at the crater terrace and at the NEC debris cone; (**b**) GBInSAR daily displacement time series of the largest displacement at the crater terrace; (**c**) GBInSAR daily displacement time series of the largest displacement at the NEC debris cone. Orange stripes identified periods characterized by daily displacements higher than 1 standard deviation of the time-series. These periods were also characterized by more frequent and stronger Strombolian explosions, and anomalous degassing^18^,^20^.

The GBInSAR system consists of a transmitting and a receiving antenna moving along a rail (3 m long in the configuration deployed at Stromboli)^1^. GBInSAR measures ground displacement along the LOS by computing, via cross correlation, the phase differences between the backscattered signals associated with two consecutive synthetic aperture radar (SAR) images. The ability of GBInSAR to measure volcano deformation depends on the persistence of phase coherence over time. The loss in coherence is primarily due to ground movements, e.g., lava flows or rock avalanches^1^,^2^. A coherence threshold equal to or above 0.8 is required to recognize deformation areas from a GBInSAR interferogram^2^. Due to the short time (11 min) between two subsequent measurements, interferometric displacements are usually smaller than half wavelength, and phase unwrapping procedures^21^ are not necessary. Both the range and cross-range resolutions are on average 2 m × 2 m, with a precision in displacement measurements of less than 1 mm^3^. Displacement rates are computed by differencing the displacements obtained from two consecutive images and dividing by the time spanned. Displacement time series (Fig. 2) are acquired using an algorithm to sum, pixel by pixel, the displacements for every consecutive pair of images and then average that rate over an 8-hour interval^3^,^22^. Displacement time series of selected points (averaged over 10 pixels) are obtained from cumulative displacement maps with a precision in the displacement measurement of 0.5 mm. Daily displacement can be calculated from the time series.

Cumulative displacement maps (Fig. 1) indicate that the NE flank of Stromboli moved upslope-downslope with the major deformation localized in the upper part of the crater terrace (Fig. 1). GBInSAR displacement time series (1 January 2010—7 August 2014) reveal fluctuations in the deformation of the summit area, with three distinct periods of inflation (July–December 2011; September 2012–May 2013; May-August 2014; orange stripes in the time series in Fig. 2), corresponding to periods of high-intensity eruptive activity, characterized by frequent and strong explosions and overflows^6^. Time series in the areas characterized by the largest displacement (Fig. 2b) reveal that the inflation of the three periods had different rates of deformation: 0.8 mm/day in July—December 2011, 1.2 mm/day in September 2012—May 2013, and 2.1 mm/day in May 2014—August 2014. The peaks in the daily displacement rate occurred on 22 August 2011, 28 March 2013 and 2 August 2014 (Fig. 2b,c). The discrete Fourier transform of the GBInSAR time series of the daily displacement of the external rim of the crater terrace (Fig. 2b), shows that the most energetic peaks correspond to a period of 256 days (Supplementary Information). We observed that the GBInSAR displacement time series of areas affected by continuous debris deposition/erosion below the NE crater (NEC) have the same trend of the upper part of the crater terrace (Fig. 2c). Cross-correlation analysis between the two daily-averaged time series reveals that the displacement fluctuation occurred simultaneously (highest absolute-value correlation 0.69 at lag time t = 0). This may mean that the either the cone of the NEC is more affected by the deformation of the conduit, regardless of the erosional/depositional processes or that the swell induced by magma in the conduit is also accompanied by depositional processes on the NEC flank.

## Modelling the Deformation

To determine the main parameters of the deformation source, we inverted the GBInSAR displacement employing the software dMODELS^23^. A number of source geometries (spherical source^24^, prolate spheroid^25^, horizontal penny-shaped source^26^ and tensile dislocation^27^), all in a flat, elastic, homogeneous, isotropic half-space are available in dMODELS^23^ for several geodetic techniques: leveling, tilt, GPS and InSAR. In the case of InSAR measurements, the software models the changes in range along the radar LOS^28^. Actual volcanic sources are not embedded cavities of simple shape but we assume that these models may reproduce the strain field created by actual storage areas and transport pathways. Given the location of the GBInSARsystem, the angle of incidence of the radar (LOS direction almost perpendicular to the volcano flank) and the fact that the system monitors only one flank of the volcano (Fig. 1), we can neglect (in first approximation) the volcano topography and model the deformation as a LOS displacement over a flat half space. The dMODELS software employs a nonlinear inversion algorithm to determine the best-fit parameters for the deformation source by searching the minimum of following the cost function^23^:


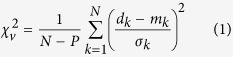


where, N is the number of data points, P the number of model parameters, *d*_*k*_ are the experimental data, *m*_*k*_ the modelling results, and *σ*_*k*_ the data uncertainties. The non-linear inversion algorithm is a combination of local optimization (interior-point method^29^) and random search. This approach is more efficient for hyper-parameter optimization than trials on a grid^30^.

We use the empirical variogram, a measure of spatial correlation^31^ to determine which one the proposed source geometries best fit the deformation (see also Table 1). When two sources would fit the data with a similar precision (e.g, the deformation episode from 08 August 2012 to 08 March 2013 can be explained by either inverse faulting or deflation of a spherical source), we choose the source with the least number of parameters^32^.

**Figure 3:**
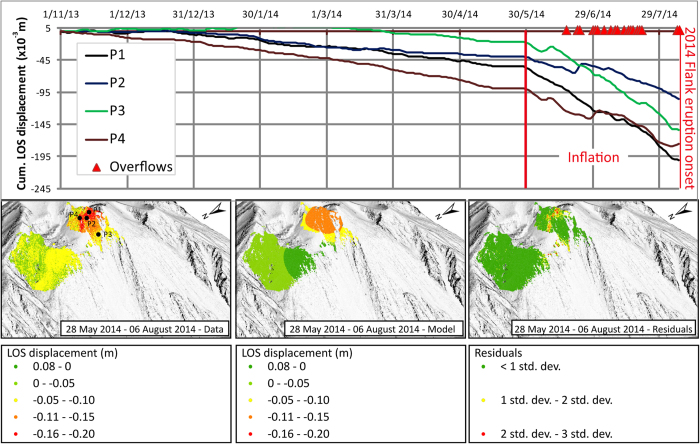
(**a**) GBInSAR time series showing the inflation in the crater terrace area since the end of May 2014, preceding the 2014 flank eruption; (**b**) Inversion results of the spherical source best-fitting the deformation between 28 May 2014—6 August 2014 (left: data; centre: model; right: residuals). The background topographic data are represented by a very high resolution Digital Elevation Model (DEM) having a spatial resolution of 50 cm provided by the “*Presidenza del Consiglio dei Ministri—Dipartimento della Protezione Civile*” (Presidency of the Council of Ministers - Department of Civil Protection) to the Dipartimento di Scienze della Terra—Università di Firenze in the framework of the research agreements SAR.net, SAR.net2, InGrID and InGrID2015 projects. This DEM was obtained elaborating the 3D data (8 pt/m^2^) acquired during the airborne laser scanning survey carried out from 04/05/2012 to 18/05/2012 by BLOM Compagnia Generale Ripreseaeree S.P.A. (www.blomasa.com). The data were acquired using the Leica ADS80 sensor which instrumental vertical and horizontal accuracy is ±10/20 cm and ±25 cm, respectively. Map was generated using ESRI 8.2^tm^ platform.

**Figure 4:**
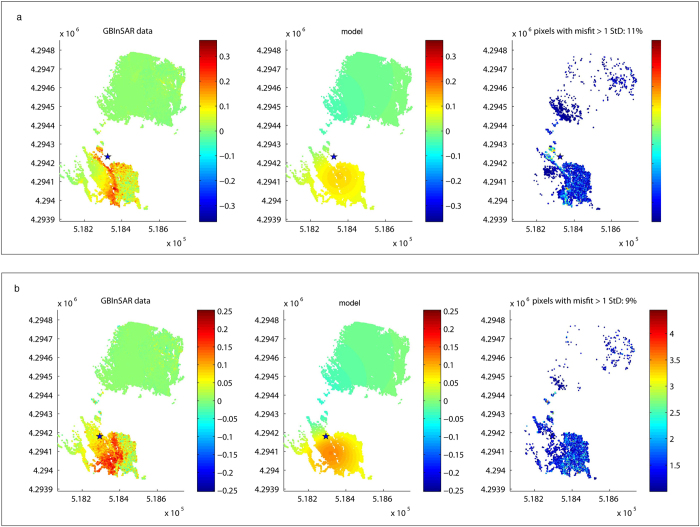
Spherical sources best fitting the deformation (see also Table 1 and Supplementary Material) - (left: data; centre: model; right: residuals). (**a**) 08 August 2012–08 March 2013; (**b**) 28 May 2014—6 August 2014; Map was generated using by dMODELS^22^.

**Figure 5:**
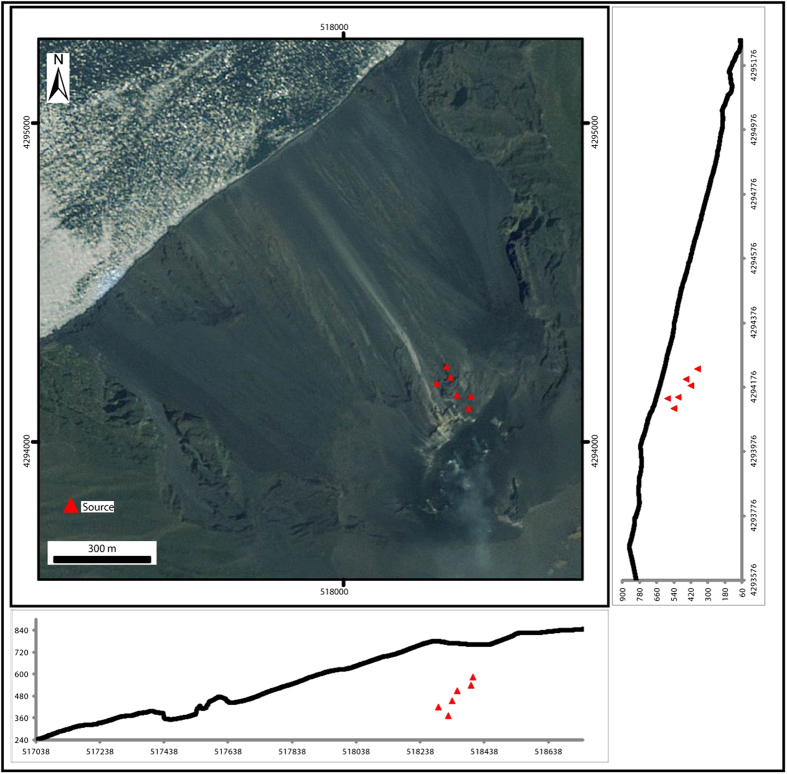
Inferred locations of the best-fit deformation source (see also Table 1). Map was generated using ESRI platform.

Examples of the inversion of InSAR measurements to determine the location of the deformation sources are shown in Fig. 3. We inverted the run-up phases for each period of high-intensity eruptive activity, choosing cumulative maps in the existing dataset (two maps per day; see Table 1). To minimize the influence from sources not related to magma accumulation, we discarded maps affected by atmospheric disturbance. The models reveal a substantial stability of the deformation source over the considered time interval. The best fitting source geometry for the 130 January 2010–August 2014 inflation is a sphere 149 ± 41 m beneath the volcano flank (Table 1; Fig. 4). The source depths can be transformed from a depth *h* relative (normal) to the volcano flank to a depth *d*_*asl*_ relative to the sea level (a.s.l.) by the simple geometric transformation


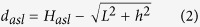


where *H*_*asl*_ is the height of the summit crater terrace above the sea level and *L* is the distance between the source surface location and the summit crater terrace. Equation (2) allowed us to estimate a source depth *d*_*asl*_ = 482 ± 46 m a.s.l. (Fig. 5).

## Discussion and Conclusive Remarks

**Figure 6:**
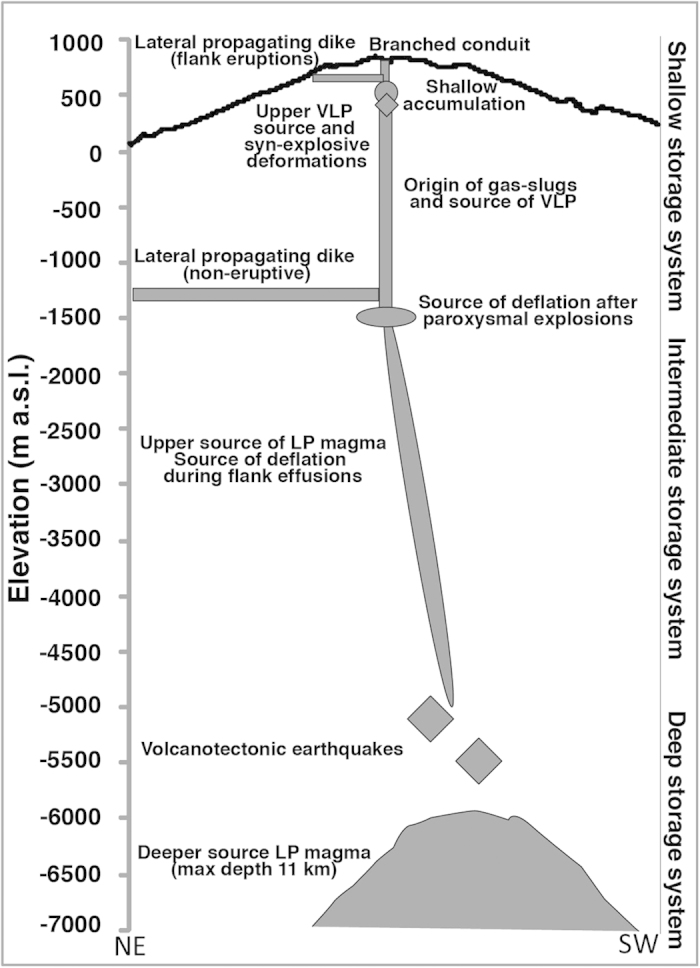
Schematic section of the Stromboli multiple-zone storage plumbing system, inferred by the integration of previous geophysical^1^,^8^,^9^,^10^,^19^,^35^,^38^, geochemical^39^,^48^,^49^ and petrological^33^,^34^,^39^,^40^ data and by results of this work. The accumulation zone identified by modelling GBInSAR data is the most shallow and likely controls the shift between summit and ephemeral (flank) eruptions.

Modelling of the deformation confirmed the presence of a very shallow reservoir consistent with the persistency of magma within Stromboli’s conduits, whose existence has been proposed before from the analysis of geochemical data^33^,^34^, that broadly corresponds with the source location of syn-explosive deformation (350–600 m a.s.l.)^35^. The presence of a very shallow reservoir has been suggested by seismic and deformation data^19^ and by petrological studies of lithic ejecta, and in particular from the evidence of pyrometamorphism in tephra accumulated within the crater terrace during persistent activity^36^. At Stromboli, zones of magma accumulation at different depth have been identified. The comparison of the source location recognized by this study with geophysical^8^,^9^,^10^,^19^,^38^, gas chemistry^39^ and petrological^33^,^34^,^39^,^40^ data argues that Stromboli’s magma plumbing configuration is a multiple-zone storage system (Fig. 6) composed by: i) a deep storage area that feed the most primitive magmas towards the surface (residence time>55 years)^40^; ii) an intermediate storage, mainly activated during energetic explosions and flank effusions (residence time = 2–10 years)^40^; iii) a shallow storage that is involved in all the surface and near-surface phenomena, including explosive activity, central and flank effusions, and non-eruptive dike injection (residence time = 10–213 days)^33^,^34^. The comparable period of the GBInSAR displacement cycles and residence time in the shallow storage system^33^,^34^ suggests that the ground displacement in the crater terrace area is controlled by the accumulation of magma in the shallow storage system.

The modelling of GBInSAR data allows us to estimate the volume of the shallow magma accumulation and to evaluate its energy budget. The January 2010 – August 2014 unrest was characterized by the accumulation of 2.76 ×  10^4^ m^3^ of magma. This value can be corrected to take into account both the effect of the magma compressibility (*β*_*m*_) and host rock (*β*_*c*_) stiffness^41^,^42^. Compressibility of basaltic magma falls in the range 0.4–2 × 10^−10^ Pa^−1^ ref. 41, while the host rock stiffness has been evaluated following the relationships:


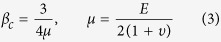


where *μ* is the shear modulus, *E* the Young’s modulus and *υ* the Poisson’s ratio. A range of values for *E* and *υ* can be obtained from ref. 43, considering both intact and damaged basalts (*β*_*c*_ in the range 0.5–1* *× 10^−10^ Pa^−1^). The corrected cumulative magma volume during January 2010—August 2014 is 4.7 ± 2.6 × 10^5 ^m^3^ (Table 1), in agreement with previous estimates (7 ± 2 10^5^ m^3^) based on geochemical data^33^,^34^. These values are also of the same order of magnitude of the volume of magma drained in the starting phase of the 2002-03 and 2007 flank eruptions^11^,^17^,^44^, suggesting that the ephemeral vents first depleted the very shallow source. The accumulation rate fluctuated over time (Table 1), with an average value of 4.4 × 10^−3^ m^3^ s^−1^ and a standard deviation of 2.5 × 10^−3^ m^3^ s^−1^. A maximum accumulation rate of 1.2 ± 0.7 ^×^  10^−2 ^m^3^ s^−1^ was estimated for the 28 May 2014-06 August 2014 inflation episode (Table 1). Based on the works of ref. 45 and ref. 46, at Stromboli 1–2 × 10^−2^ km^3^ year^−1^ of magma is degassed, of which only ≈7 × 10^−5^ km^3^ year^−1^ is erupted^47^. Our calculation would account for 1.5 ± 0.8 × 10^−4^ km^3^ year^−1^ of magma stored in the upper portion of the volcano. This leads to the conclusion that the larger portion of the magma fed at the surface falls back in the conduit due to convection^39^,^48^,^49^. Using the model proposed in ref. 50, we evaluated that the very shallow source recognized in this work to remain molten and open at a very shallow depth, need heat to its margins at a rate of <1 MW, much lower than heat release at the crater terrace (423 ± 226 MW) evaluated by ref. 46.

**Figure 7:**
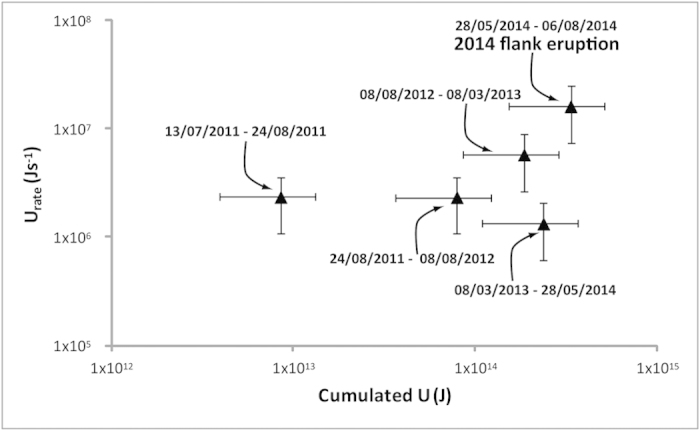
Diagram showing potential energy stored vs potential energy rate (see also Table 1). The maximum rate and strain energy accumulated at Stromboli coincide with the flank eruption of August 2014.

The 2014 Stromboli flank eruption was characterized by the development of an eruptive fissure that moved the eruptive centre from the summit crater terrace at 750 m a.s.l. to an ephemeral vent at 650 m a.s.l. The initiation and propagation of a fracture depend primarily on a first order on the potential energy stored in a volcanic edifice when it is loaded^51^,^52^. Here the loading is primarily related to inflation of the shallow magma storage area. We calculate the strain energy of the shallow intrusions using the strain-nucleus *U*_*n*_^52^


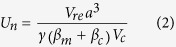


where *V*_*re*_ is the volume of magma in a single deformation episode, *V*_*c*_ is the total magma volume, assumed here as the volume accumulated during the previous time intervals, *a* is the magma body radius and *γ* is the melt fraction (on average 50%)^39^,^53^. The strain energy in each analyzed interval is presented in Table 1. The total strain energy stored during the period January 2010—August 2014 was *U*_*n*_ = 3.3 ± 1.8 × 10^14^ J (Table 1). The surface energy needed to open the 6–7 August eruptive fissure, considering a 170 m long (strike dimension, planar distance between the NE crater and the ephemeral vent) and 100 m tall (dip dimension, high difference between the NE crater and the ephemeral vent) fracture and an energy density of about 1.3–43 × 10^6^ J m^−2^ (see ref. 51), is in the range of 10^9^–10^11^ J, much lower than the total energy stored at Stromboli during the January 2010—August 2014 inflation. The maximum rate and strain energy accumulated at Stromboli coincide with the inflation preceding the flank eruption of August 2014 (Fig. 7).

Ground displacement in the crater terrace area is controlled by the accumulation of magma in the shallow storage system. Fracture opening and propagation is controlled by an increase in the magma accumulation rate that allows building up significant potential energy in a short amount of time. Variations in GBInSAR time series reflect variation in strain energy stored in the shallow source and could be used to forecast the shift between summit and flank eruptions.

## Additional Information

**How to cite this article**: Di Traglia, F. *et al.* Shifts in the eruptive styles at Stromboli in 2010–2014 revealed by ground-based InSAR data. *Sci. Rep.* **5**, 13569; doi: 10.1038/srep13569 (2015).

## Supplementary Material

Supplementary Information

## Figures and Tables

**Table 1:** Inversion statistics, inversion results, volume and energy changes−*σ* is the standard deviation.

Inversion statistics							
Interval	Pixels	%pixelsmisfit < 1 σ	%pixelsmisfit < 2 σ	Nugget model	R2		VariogramNRMSE
01/01/2010—13/07/2011	80892	91%	97%	0.011 ± 0.008	0.732		0.125
13/07/2011—24/08/2011	80892	82%	93%	0.012 ± 0.007	0.714		0.431
24/08/2011—08/08/2012	80890	85%	95%	0.008 ± 0.006	0.742		0.175
08/08/2012—08/03/2013	80892	89%	98%	0.008 ± 0.006	0.769		0.149
08/03/2013—28/05/2014	80892	91%	97%	0.015 ± 0.012	0.654		0.129
28/05/2014—06/08/2014	80892	91%	98%	0.009 ± 0.006	0.748		0.094
Inversion results (see also Fig. 4 and Fig. 5)							
Interval	x0 (m)	y0 (m)	z0 (m)	dV (m^3^)	Sourcedepth belowcrater (m)	Radius (m)	Sourcealtitude(m a.s.l.)
01/01/2010—13/07/2011	518353	4294144	139	6.71 × 10^4^	−154	32	596
13/07/2011—24/08/2011	518337	4294200	152	2.85 × 10^3^	−168	32	582
24/08/2011—08/08/2012	518396	4294109	142	2.36 × 10^4^	−157	32	593
08/08/2012—08/03/2013	518325	4294232	204	3.54 × 10^4^	−226	32	524
08/03/2013—28/05/2014	518402	4294140	82	1.72 × 10^4^	−91	32	659
28/05/2014—06/08/2014	518294	4294180	177	2.76 × 10^4^	−196	32	554
Volume changes taking into account magma compressibility							
Interval	Av. injectedvolume foreach episode(m^3^)	σ	Av. injectedcumulated volume2010–2014(m^3^)	σ	Accumulation rate foreach episode (m^3^ s^−1^)		σ
01/01/2010—13/07/2011	1.82 × 10^5^	1.01 × 10^5^	1.82 × 10^5^	1.01 × 10^5^	3.77 × 10^−3^		2.10 × 10^−3^
13/07/2011—24/08/2011	7.72 × 10^3^	4.29 × 10^3^	1.90 × 10^5^	1.05 × 10^5^	2.13 × 10^−3^		1.18 × 10^−3^
24/08/2011—08/08/2012	6.40 × 10^4^	3.56 × 10^4^	2.54 × 10^5^	1.412 × 10^5^	2.12 × 10^−3^		1.18 × 10^−3^
08/08/2012—08/03/2013	9.61 × 10^4^	5.34 × 10^4^	3.50 × 10^5^	1.94 × 10^5^	5.25 × 10^−3^		2.92 × 10^−3^
08/03/2013—28/05/2014	4.65 × 10^4^	2.59 × 10^4^	3.96 × 10^5^	2.20 × 10^5^	1.21 × 10^−3^		6.71 × 10^−4^
28/05/2014—06/08/2014	7.48 × 10^4^	4.16 × 10^4^	4.71 × 10^5^	2.62 × 10^5^	1.24 × 10^−2^		6.88 × 10^−2^
Energy changes							
Interval	Av. energy foreach episode(J)	σ	Av. cumulatedenergy2010 – 2014 (J)	σ	Energy rate for eachepisode (J s^−1^)		σ
13/07/2011—24/08/2011	8.51 × 10^13^	4.60 × 10^12^	8.51 × 10^12^	4.60 × 10^13^	2.34 × 10^6^		1.27 × 10^6^
24/08/2011—08/08/2012	7.05 × 10^13^	3.81 × 10^13^	7.90 × 10^13^	4.27 × 10^13^	2.33 × 10^6^		1.26 × 10^6^
08/08/2012—08/03/2013	1.06 × 10^13^	5.72 × 10^13^	1.85 × 10^14^	9.99 × 10^13^	5.78 × 10^6^		3.12 × 10^6^
08/03/2013—28/05/2014	5.13 × 10^13^	2.77 × 10^13^	2.36 × 10^14^	1.28 × 10^14^	1.33 × 10^6^		7.19 × 10^6^
28/05/2014—06/08/2014	9.74 × 10^13^	5.26 × 10^13^	3.34 × 10^14^	1.80 × 10^14^	1.61 × 10^7^		8.70 × 10^6^

Inversion statistics: the nugget model for a data set with no significant spatial correlation should be zero within two standard deviations. R2 is a measure of the percentage of data explained by the model; the variagram NRMS is a measure of how good is the comparison between the experimental and model variogram. Inversion results: the source altitude above sea level is given by (2). Inversion results: x0, y0 and z0 are the modelling results, z0 is the depth normal to the volcano flank.
